# Consequences of United States funding suspensions on community‐led HIV services in Latin America and the Caribbean: findings of a rapid service provider survey

**DOI:** 10.1002/jia2.70081

**Published:** 2026-02-10

**Authors:** José Rafael Guillén, Megan Stevenson, Miguel Ángel Barriga Talero, Mary Ann Torres, Andrea L. Wirtz

**Affiliations:** ^1^ Red Somos Bogotá Colombia; ^2^ Department of Epidemiology Center for Public Health and Human Rights, Johns Hopkins University Bloomberg School of Public Health Baltimore Maryland USA; ^3^ International Council of Aids Service Organizations (ICASO) Toronto Ontario Canada; ^4^ Department of International Health Center for Humanitarian Health, Johns Hopkins School of Public Health Baltimore Maryland USA

**Keywords:** Caribbean, community‐based, financing, HIV, Latin America, PEPFAR, USAID

## Abstract

**Introduction:**

Foreign aid provided by the United States government (USG), including support for HIV services, has been suspended or dismantled since January 2025. Early research and modelling projections have suggested significant impacts globally and in sub‐Saharan Africa. We aimed to evaluate the consequences of USG funding suspensions on community‐led HIV services in Latin America and the Caribbean (LAC).

**Methods:**

We surveyed community‐led organizations providing HIV services in LAC in February−March 2025. Organization leaders were recruited through a network of HIV service organizations. Survey items measured USG funding (past 12 months) and funding sources; experiences of funding suspensions; and programmes, beneficiaries and workforce affected by funding suspensions.

**Results:**

Of 40 respondent organizations, 24 (60%) had received USG funding in the past 12 months. Of the 24, 21 (87%) organizations representing 10 countries reported that they had experienced a funding suspension. These included direct and indirect funding from PEPFAR (62%), USAID (48%) and, less frequently, other USG agencies. Programmes most frequently affected by the funding freeze included sexual prevention programmes, HIV testing services, psychosocial support and humanitarian services. An estimated 156,164 beneficiaries had been receiving HIV services across respondent organizations that were affected by funding suspensions. Populations commonly served included (but were not limited to) people living with HIV, transgender people, people engaged in sex work, men who have sex with men, migrant populations and women.

**Conclusions:**

LAC HIV programmes have had comparatively less reliance on USG funding than other regions; however, they are still likely to be significantly affected by funding suspensions and the dismantling of USAID. Achievement of global HIV goals in LAC will require support from government and foreign donors, as well as collaboration with pharmaceutical companies to ensure access to biomedical HIV prevention and treatment.

## 1 INTRODUCTION

Since the start of the epidemic, the Latin American region has generally had low HIV prevalence and incidence and was considered to have a concentrated HIV epidemic [1]. Consequently, this led to a lower prioritization of global HIV response in the region and recent increases in new HIV acquisitions. In 2024, the UNAIDS global report indicated that the Latin American region was one of only three regions globally in which the number of new acquisitions increased since 2010 [2, 3]. UNAIDS further reported that the number of acquisitions was approximately 20% higher in 2022 than in 2010 among gay men and other men who have sex with men (MSM), 20% higher among transgender women and 42% higher among sex workers [3]. The number of AIDS‐related deaths also increased among women in Paraguay, Peru, Costa Rica, El Salvador, Mexico, and Panama despite observed declines in most countries globally [3].

The epidemiology is different in the Caribbean, where HIV prevalence has been higher [3]. In Haiti, for example, HIV prevalence peaked at 2.7% among adults aged 15–49 years in 1995 [4]. Consequently, the Caribbean received greater investment from the U.S. President's Emergency Plan for AIDS Relief (PEPFAR) and other foreign support and ultimately observed 22% decline of new acquisitions between 2010 and 2023 in the region [3].

Latin America has been relatively less dependent on foreign funding than other regions of the world, especially with respect to testing and treatment [5, 6]. However, PEPFAR's Western Hemisphere regional programme provided financial support for HIV treatment programmes as well as more recent programmes to support HIV prevention, including pre‐exposure prophylaxis (PrEP) [7]. The Western Hemisphere PEPFAR planned allocation for fiscal year 2024 included support for Brazil, Colombia, Guatemala, Honduras, Jamaica, Nicaragua, Panama, Peru, El Salvador, and Trinidad and Tobago, which were predominantly administered by USAID, the U.S. Centers for Disease Control and Prevention (CDC) and other U.S. agencies [7]. Greater funding was provided to the Caribbean and PEPFAR country programmes included Haiti and the Dominican Republic [8]. Argentina, Brazil, Chile, Colombia, Mexico and Uruguay constitute the only countries in the region whose national HIV programmes are not directly dependent on donor funding; however, even in these countries, many non‐governmental organizations (NGOs) and private clinics still rely on donor funds, many of which serve the most affected populations [9].

HIV PrEP and other prevention programmes in Latin America and the Caribbean (LAC) countries are reliant to a heavier degree on foreign funding than treatment and testing programmes [6, 10]. Consequently, PrEP programmes are nascent in many LAC countries. A 2023 review reported that of 33 countries in LAC, only 22 had PrEP policies and 13 had incorporated PrEP into the country's national health system [11], though more recent data from PrEPwatch.org indicate these numbers are slowly increasing [12]. Only two countries in LAC—Peru and Brazil—have approved long‐acting injectable PrEP (Cabotegravir) in conjunction with their participation in the clinical trial [12, 13], despite preference for this modality among several populations who can most benefit from HIV prevention services in Latin America [11, 14, 15].

Changes in U.S. federal priorities in early 2025 have led to funding suspensions and terminations and threatened the continuation of HIV programmes globally. On 20 January 2025, the U.S. government (USG) announced a 90‐day pause on all foreign assistance, including PEPFAR [16, 17]. Though a waiver was issued 8 days later permitting the resumption of life‐saving medicines and medical services, the confusion related to what services were covered led to many programmes remaining shuttered [16]. Subsequently, the USG terminated their agreement with UNAIDS on 27 February and announced the closure of USAID, which administers PEPFAR funds in many LAC countries, on 28 March 2025 [16]. PEPFAR alone was a major contributor to the global HIV response, among many services, providing treatment for an estimated 20.6 million people living with HIV and supporting PrEP initiations among 2.5 million people as of 2024 [18]. Modelling projections of 26 countries globally estimated that the loss of PEPFAR, coupled with announced reductions from four other major donor countries, could result in an additional 4.43–10.75 million new HIV acquisitions and 0.77–2.93 million HIV‐related deaths between 2025 and 2030 [19]. Even if the PEPFAR freeze were reversed after the 90‐day pause, over 100, 000 excess HIV deaths per year were predicted in sub‐Saharan Africa alone [20]. While recent analyses have sought to estimate the impact of these funding suspensions globally and in sub‐Saharan Africa [20, 21, 22, 23], few have explored the potential impact in LAC. We conducted a rapid survey to assess the direct consequences of USG foreign funding suspensions on community‐led HIV services in LAC.

## 2 METHODS

The study was conducted in close partnership between Red Somos, a Colombian community‐based organization, ICASO, an advocacy organization and Johns Hopkins Bloomberg School of Public Health, a research institution, with a collaborative history in HIV research.

The survey was developed in English, Spanish, Portuguese, and Haitian Creole and reviewed by representatives from two partner community‐based HIV service organizations prior to distribution. Survey items collected information on organization characteristics, including type, country location, number of offices, and receipt of USG funding in 2024–2025 and source (direct and indirect from PEPFAR, USAID, NIH, CDC or other). For those reporting receipt of USG funding, other items measured whether funding had been suspended, funding level and proportion of operational budget, HIV services affected by suspensions, population groups and number of adult and children beneficiaries receiving affected services, and whether services were expected to continue in any capacity beyond the suspensions.

Data collection spanned 18 February–14 March 2025. The electronic survey was circulated through community networks of HIV service organizations spanning the LAC region. One leader with knowledge of programme funding from each organization was asked to complete the survey. Respondents were asked to provide the organization name for identification and removal of potential duplicate entries; this was a voluntary field, given the sensitive nature of the data. The organization names were confidentially stored and only accessed by authors working directly with the data.

We calculated descriptive frequencies of organization characteristics and experiences of funding suspensions. We summed the number of adult (aged 18 and older), children (aged 17 and younger) and total beneficiaries; budgets; and staff terminations associated with funding suspensions across organizations and calculated the median and interquartile ranges (IQR) per organization.

The study was reviewed by the Johns Hopkins Bloomberg School of Public Health Institutional Review Board, which determined the study was “Not Human Subjects Research.” This determination was because the survey was for organizational leaders and inquired only about funding and services provided by their organization. Because of the determination, a consent form was not required; however, we provided information about the intent of the study, topic of the survey, and potential risks to and data protections; respondents were required to read through this information and acknowledge before initiating the survey questionnaire.

## 3 RESULTS

Data collection spanned 18 February–14 March 2025. After eliminating forms with invalid fields and duplicate entries, 40 organizations provided information for this analysis.

Participants described their organizations as NGOs or community‐based organizations, and one international NGO. Respondent organizations represented 13 countries including: Bolivia, Colombia, Costa Rica, Dominican Republic, Ecuador, Guatemala, Honduras, Haiti, Panama, Paraguay, El Salvador, Trinidad and Tobago, and Venezuela. Participating organizations reported a median of one office/clinic (IQR: 1–3) per country. Of 40 respondents, 24 (60%) reported that they had received funding from the USG in the past 12 months. Sources of funding typically included USAID through another organization, direct funding from USAID and PEPFAR through another organization or entity. Of the 24 with USG‐funded programmes, 21 (87%) reported that they had received a suspension or funding freeze, two had not and one preferred not to answer.

**Figure 1:**
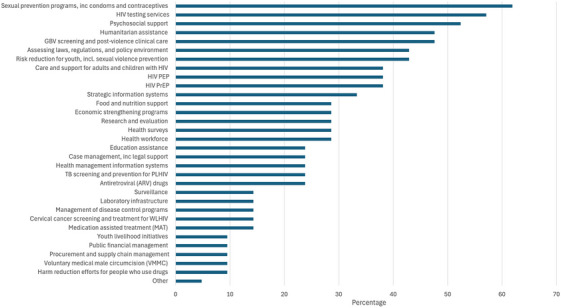
Types and percentages of services affected by 2025 U.S. government funding suspensions in Latin America and the Caribbean among respondent organizations with funding suspensions (*n* = 21). *Note*: Percentages do not sum to 100 due to multiple allowed responses regarding types of services provided; among organizations in countries with PrEP approved, 44% had their services affected by suspensions. GBV, gender‐based violence; PEP, post‐exposure prophylaxis; PLHIV, people living with HIV; PrEP, pre‐exposure prophylaxis; TB, tuberculosis; WLHIV, women living with HIV.

The 21 organizations with funding freezes represented 10 countries: Bolivia, Colombia, Costa Rica, Dominican Republic, Ecuador, Honduras, Haiti, Panama, El Salvador and Venezuela. Table 1 describes the experiences of the 21 organizations with funding freezes. Affected funding sources included direct and indirect funding from PEPFAR (62%), direct and indirect funding from USAID (48%) and, less frequently, direct and indirect funding from the NIH, CDC or the U.S. State Department (48%). The median annual budget suspended was USD$140, 000 per organization (IQR: $87, 500−343, 727) and totalled $8, 348, 154 across the organizations. This represented a median 46% of the organizations’ annual budgets (IQR: 25–85), though reached 100% for two organizations. Thirty‐two types of programmes were affected by the funding freeze, but most commonly included sexual prevention programmes, HIV testing services, psychosocial support, humanitarian services, and GBV prevention and clinical care (Figure 1). Thirty‐eight percent of the affected programmes provided HIV PrEP and post‐exposure prophylaxis (PEP); however, PrEP services represented 44% (8/18) of affected programmes when restricting to organizations in countries where PrEP has been approved.

**Table 1:** Characteristics of respondent organizations affected by U.S. government funding suspensions in Latin America and Caribbean as of March 2025 (*n* = 21)

	*n*	Percent (%)
**Funding source that received stop work order (multiple responses allowed)**		
Direct funding from PEPFAR	2	9.5
PEPFAR through USAID	3	14.3
PEPFAR through another organization	8	38.1
Direct funding from USAID	4	19
USAID through another organization	6	28.6
Direct funding from U.S. CDC	1	4.8
CDC through another organization	3	14.3
Direct funding from U.S. NIH	1	4.8
NIH through another organization	2	9.5
Other, specify	3	14.3
**Affected countries**		
Bolivia	1	4.76
Colombia	2	9.52
Dominican Republic	2	9.52
Ecuador	3	14.29
Guatemala	3	14.29
Honduras	1	4.76
Haiti	4	19.05
Panama	2	9.52
El Salvador	1	4.76
Venezuela	2	9.52
**Median number of offices in affected programmes (per country)**	2	(1‐3)
**Median annual budget in programmes that were stopped ($USD), IQR**	$140, 000	($87, 500−343, 727)
***Total budget stopped in region***	*$8, 348, 154*	
**Median percent of organization annual budget (IQR)**	46	(25−85)
**Median number of adults living with HIV served per organization (IQR)**	193	(69−400)
***Total adults living with HIV served***	*16, 179*	
**Median number of children with HIV served per organization (IQR)**	0	(0−10)
***Total number of children with HIV served***	*1270*	
**Median number of adults without HIV or status unknown per organization (IQR)**	2500	(150−5203)
***Total adults without HIV or status unknown***	*117, 513*	
**Median children not living with HIV or status unknown per organization**	5	(0−50)
***Total children served***:	*21, 202*	
***Total people served***:	*156, 164*	
**Terminated any staff or contractors because of stop work order (*n* = 18)**		
No	1	5.6
Yes	15	83.3
Unsure/prefer not to answer	2	11.1
**Median number of staff terminated**	10	(4−20)
***Total terminated***	***178***	
**Programme(s) can continue without this funding (*n* = 18)**		
Not at all	10	55.6
Some may continue	6	33.3
Unsure/prefer not to answer	2	11.1
**If funding were restored, programme(s) could resume at the same level**		
Yes, but would be reduced	5	27.8
Yes, entirely	13	72.2
**Any other U.S.‐funded programmes continued (*n* = 21)**		
No	15	71.4
Yes	1	4.8
Not applicable	4	19
Unsure/prefer not to answer	1	4.8

Abbreviations: IQR, interquartile range; NIH, National Institute of Health; PEPFAR, President's Emergency Plan for AIDS Relief; USAID, U.S. Agency for International Development; U.S. CDC, U.S. Centers for Disease Control and Prevention; USD, U.S. Dollar.

**Figure 2:**
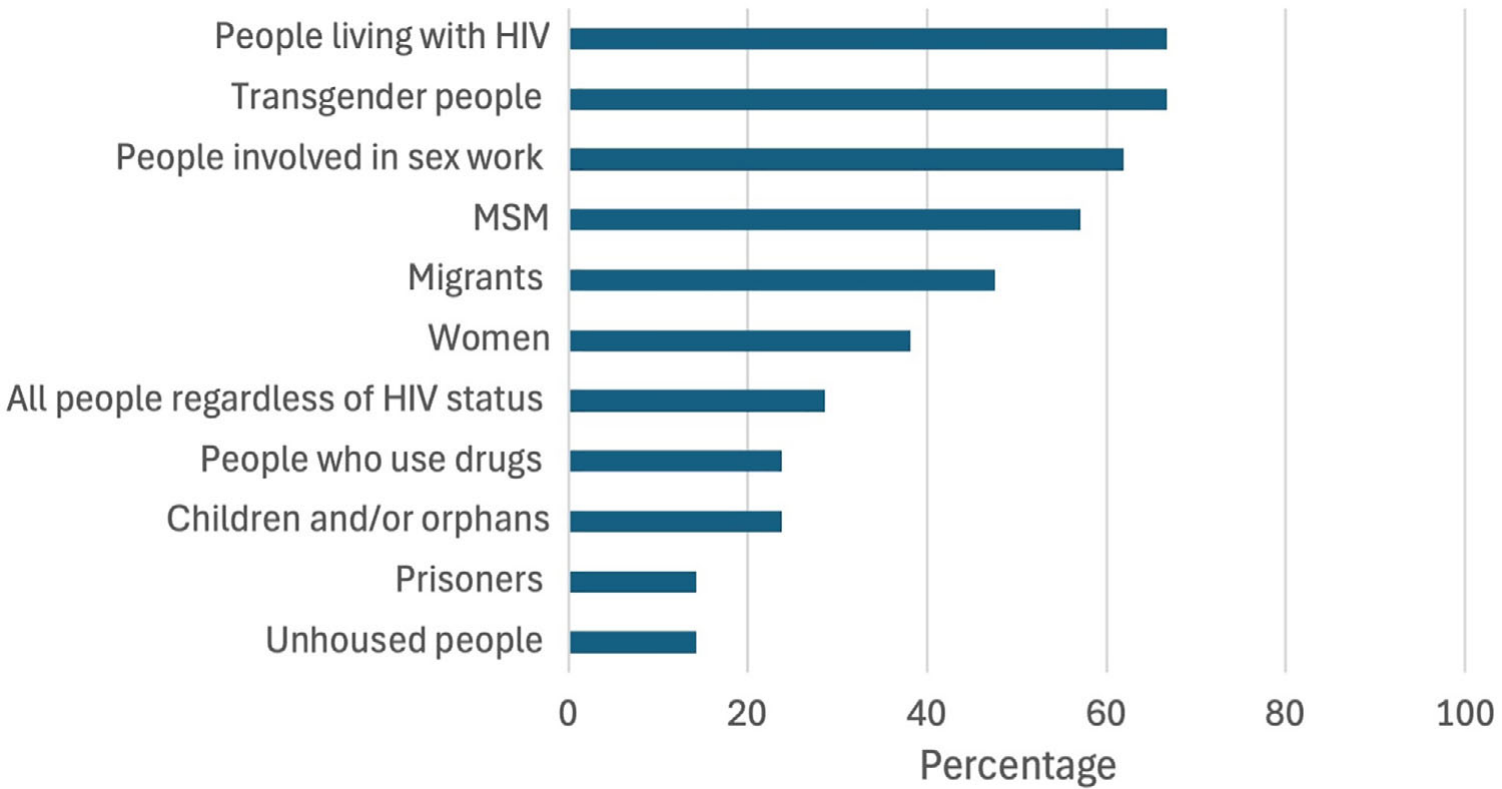
Recipient groups of services that were affected by 2025 U.S. government funding suspensions in Latin America and the Caribbean among respondent organizations with funding suspensions (*n* = 21). *Note*: Percentages do not sum to 100 due to multiple allowed responses regarding recipient groups.

Affected programmes served a median of 193 adults living with HIV per organization (IQR: 69–400), totalling 16, 179 adults, and served a median of 0 children living with HIV per organization (IQR: 0–10), totalling 1270 across organizations. Additionally, organizations served a median of 2500 adults without HIV or with status unknown per organization (IQR: 150–5203), totalling 117, 513 adults, and a median of 5 children without HIV or status unknown (IQR: 0–50), totalling 21, 202 children. In total, 156, 164 beneficiaries had been receiving HIV services that were affected by funding suspensions. Populations commonly served by these programmes included (but were not limited to) people living with HIV, key populations including transgender people, people engaged in sex work, gay men and other men who have sex with men, migrant populations and women (Figure 2).

Funding freezes affected the organizations’ staff workforce. Of 18 respondent organizations, 13 (83%) reported that they were forced to terminate staff positions or contractors, and two were uncertain. A median of 10 staff per organization were terminated (IQR: 4–20), including a maximum of 32 in one organization, totalling 178 staff members or contractors across the affected organizations. Over half of affected organizations (10/18; 56%) reported that they absolutely could not continue the programmes without the funding that had been suspended; six (33%) reported that some of the programmes could continue and two were unsure. If funding was to be restored, 13 (72%) felt that the programmes could resume at their original scale, and five (28%) reported that they could resume but at reduced levels.

Only two organizations with USG funding reported that their funding had not been suspended (a third respondent was unsure). One of these two organizations did, however, receive reductions to their funding, was required to revise or remove certain public‐facing information and was told they could not serve certain populations.

In open text fields, several organizations voiced concerns about the security of their programmes, beneficiaries and offices, given the lack of governmental support in many of their countries. Some organizations that did not receive USG funding in the past 12 months reported receiving funds instead through the Global Fund to Fight AIDS, TB, and Malaria and expressed concern that the withdrawal of the U.S. financial support from multilateral financing mechanisms would adversely impact the remaining programmes in the region.

## 4 DISCUSSION

In this rapid survey of community‐led HIV service organizations in LAC in early 2025, we observed that 60% of participating organizations had been supported through USG funds in the past year and, of these, 87% had their funding suspended. Ultimately, we estimated that among the respondent organizations, a total of 156, 164 beneficiaries may lose access to HIV services because of funding suspensions experienced by respondent organizations alone. These findings suggest that while HIV programmes in LAC have had less reliance on USG health financing, they are nonetheless likely to be significantly impacted as the future of USG HIV funding is unclear. A July 2025 rescissions package was approved by the USG to rescind almost USD$8 billion in foreign aid programmes [24]. Though USD$400 million designated for PEPFAR was spared from the cuts and offered some hope [24], it is likely that the cuts to humanitarian programmes will have downstream impacts for countries that are supporting displaced populations. Further, a draft plan by the U.S. Department of State mapped out a dissolution of the PEPFAR programme in the coming years [25].

Because 48% of suspended funding was reported to come directly and indirectly from USAID, the subsequent dismantling of USAID after the implementation of this study raises questions about the future of USG‐funded programmes in LAC, even if suspended PEPFAR programmes were to be restored. A recent evaluation of USAID programming supports such concerns. Cavalcanti and colleagues estimated that USAID interventions implemented globally between 2000 and 2021 were responsible for preventing 91, 839, 663 all‐age deaths, including 25.5 million deaths associated with HIV; however, their forecasting models predicted that the current termination of USAID could result in more than 14, 051, 750 additional all‐age deaths by 2030 [23]. One organization did not have NIH funding suspended but was requested to change public‐facing information and remove provision of services to certain populations, as many other NIH‐funded researchers were required to do if their programme focused on gender, race or country of origin [26, 27]. Respondent organizations that had reported they had not received USG funding, nonetheless, also expressed concerns in qualitative fields about the future of programmes funded by the Global Fund to Fight AIDS, TB, and Malaria. Their concerns were based on the knowledge that the United States was the leading donor to the Global Fund, providing almost one‐third of the Fund's budget [28], which provides financial support to many LAC countries, including both government and non‐governmental organizations [29]. If intentions stated by the administration to reduce U.S. contributions to the Global Fund are realized [30], these will likely further disrupt HIV and related public health programmes in LAC and globally.

USG funding suspensions are likely to reduce access to care for people living with HIV and may result in worsening HIV outcomes. We estimated that among respondent organizations alone, 16, 179 adults and 1270 children living with HIV will be impacted, though this reflects only a small fraction of the people living with HIV in LAC who have been and will continue to be impacted by the suspensions. UNAIDS has reported that 60% of Haiti's national HIV response, including treatment for 127, 000 PLHIV, was supported by PEPFAR [31]. The impacts on HIV care are exacerbated by impacts on clinical services, the supply chain and logistics. For example, a May 2025 HIV Commodities report indicated that Haiti experienced a shortage of at least one ARV line in the past 2 months and had less than a 6‐month supply of two paediatric ARV lines [32]. These challenges are likely to exacerbate declining access to care, as they follow the closure of 39% of healthcare facilities, including two major hospitals, due to escalating violence in the country [31]. Similarly, one‐third of the Dominican Republic's HIV care services were supported through PEPFAR; the suspensions are expected to leave thousands without access to essential HIV services, despite the fact that the government guarantees access to HIV treatment [31].

Any recent progress on the HIV epidemic in LAC made through the introduction of PrEP is also likely to be drastically affected, particularly for government programmes that relied on PEPFAR and USAID funding. A significant proportion of services affected by funding suspensions reported in this survey supported HIV testing and prevention, as well as other ancillary services that aim to support access and adherence to prevention and treatment. These findings are supported by government commodities reports, which indicated that El Salvador and Guatemala had less than 6 months of stock of HIV test and viral load supplies, respectively, as of May 2025 [32]. We estimated that 117, 513 adults and 2120 children who were receiving HIV prevention services through respondent organizations have had these services disrupted, and many respondent organizations were pessimistic that the programmes would be continued with the support of their government.

Beneficiary populations affected by funding suspensions were predominantly serving LGBTQ populations and migrants—groups that are both disproportionately impacted by HIV in the region but also deprioritized and often targeted by the current U.S. administration [33, 34, 35]. UNAIDS reported a rippling effect in the LAC region following changes in USG policy, describing intensifying homophobia, transphobia and discrimination against PLHIV in several countries in LAC and noting that parliamentarians in the region have taken advantage of this situation to gain political leverage by attacking minoritized populations [36]. The same report points out that loss of non‐HIV funds, or even non‐public health funds, deepens vulnerabilities of those most at risk for HIV acquisition, such as those affected by conflict in Colombia, refugees and migrants, and those impacted by humanitarian crises in Haiti and Venezuela, by putting them at increased risk of exploitation and human trafficking [36].

This study has limitations. First, because this was a rapid survey of 40 HIV service organizations, our findings do not capture the number of people affected by funding suspensions among LAC organizations who did not participate in this survey; thus, respondent organizations likely represent a small fraction of affected USG funding recipients in LAC. Further, as a rapid survey conducted in February through March 2025 following the first round of suspensions, we would not have ascertained any suspensions or reinstatements that occurred after March, including the dismantling of USAID, terminations of NIH grants that focused on LGBTQ populations, women, migrants or were suspended foreign subcontracts [26, 27]. Data collection was implemented during a time of confusion and uncertainty for funding recipients, which may have impacted the validity of the study results.

## 5 CONCLUSIONS

Our findings add to and expand upon the emerging literature by describing the impact of the funding suspensions on organizations providing HIV services in LAC. Specifically, this manuscript describes important patterns in how changes in USG funding may affect HIV services, particularly prevention, in the region, but also highlights resilience among community‐led programmes to continue HIV services in these conditions. Foreign donors who may work to fill gaps in funding are encouraged not to ignore the epidemic in LAC. Other solutions, such as drug licensing agreements, are urgently needed to assure access to highly effective HIV prevention and treatment medicines in LAC.

## COMPETING INTERESTS

This study was not funded and was conducted through voluntary work by the authors. Preliminary study findings have been presented at the 2025 International AIDS Society Conference in Rwanda. ALW and MS receive unrelated research funding to their institution from ViiV Healthcare. While this manuscript was under review, ALW became an employee of Gilead Sciences and owns shares in Gilead.

## AUTHOR CONTRIBUTIONS

ALW, MS, JRG and MABT conceived of and designed the study; ALW and MS drafted the survey instruments with input from JRG, MABT and MAT; JRG, MABT and MAT recruited participants to the study; ALW and MS oversaw the implementation of the electronic survey and ALW conducted the analysis; ALW and MS wrote the first draft of the manuscript with review and input by MABT, JRG and MAT. All authors reviewed and approved the manuscript for publication.

## Data Availability

The data that support the findings of this study are available from the corresponding author upon reasonable request.

## References

[1] Luz PM , Veloso VG , Grinsztejn B . The HIV epidemic in Latin America: accomplishments and challenges on treatment and prevention. Curr Opin HIV AIDS. 2019;14(5):366–73. 31219888 10.1097/COH.0000000000000564 PMC6688714

[2] UNAIDS . 2025 Global AIDS Update—AIDS, Crisis, and the Power to Transform: Latin America—Regional Profile. 2025.

[3] UNAIDS . The urgency of now: AIDS at a crossroads. 2024. https://www.unaids.org/sites/default/files/media_asset/2024‐unaids‐global‐aids‐update_en.pdf.

[4] World Bank Group . Prevalence of HIV, total (% of population ages 15–49)—Haiti. 2025. accessed December 1, 2025. https://data.worldbank.org/indicator/SH.DYN.AIDS.ZS?locations=HT

[5] UNAIDS . The response to HIV in Latin America—Global AIDS update 2019. 2019.

[6] Arán‐Matero D , Amico P , Arán‐Fernandez C , Gobet B , Izazola‐Licea JA , Avila‐Figueroa C . Levels of spending and resource allocation to HIV programs and services in Latin America and the Caribbean. PLoS One. 2011;6(7):e22373. 21799839 10.1371/journal.pone.0022373 PMC3142155

[7] U.S. Global AIDS Coordinator . Fiscal Year (FY) 2024 PEPFAR Planned Allocation: Western Hemisphere Region. 2023. https://www.state.gov/wp‐content/uploads/2023/02/Western‐Hemisphere‐ROP23‐PLL_02_15_2023.pdf.

[8] US Department of State . Where We Work—PEPFAR: Country Operational Plans. accessed July 23, 2025. https://www.state.gov/where‐we‐work‐pepfar

[9] Luz PM , Veloso VG , Grinsztejn B . The HIV epidemic in Latin America: accomplishments and challenges on treatment and prevention. Curr Opin HIV AIDS. 2019;14(5):366–73. 31219888 10.1097/COH.0000000000000564 PMC6688714

[10] UNAIDS . An Evaluation of UNAIDS Joint Programme Country Envelopes: 2018–2022. 2023. accessed October 27, 2025. https://www.unaids.org/sites/default/files/media/documents/evaluation‐country‐envelopes‐2018‐2022‐case‐study‐andean_en.pdf

[11] Murphy L , Bowra A , Adams E , Cabello R , Clark JL , Konda K , et al. PrEP policy implementation gaps and opportunities in Latin America and the Caribbean: a scoping review. Ther Adv Infect Dis. 2023;10:20499361231164030. 10.1177/20499361231164030 37114192 PMC10126665

[12] AVAC . Global PrEP Tracker: PrEP Regulatory Approvals by Year. 2024. accessed July 16, 2025. https://data.prepwatch.org/

[13] Landovitz RJ , Donnell D , Clement ME , Hanscom B , Cottle L , Coelho L , et al. Cabotegravir for HIV prevention in cisgender men and transgender women. N Engl J Med. 2021;385(7):595–608. 34379922 10.1056/NEJMoa2101016 PMC8448593

[14] Sciannameo S , Zalazar V , Spadaccini L , Duarte M , Cahn P , Aristegui I , et al. Preference for long‐acting injectable for ART and PrEP among people with and without HIV: a cross‐sectional study in Argentina. Ther Adv Infect Dis. 2024;11:20499361241228341. 10.1177/20499361241228341 38380160 PMC10878205

[15] Torres TS , Nascimento AR , Coelho LE , Konda KA , Vega‐Ramirez EH , Elorreaga OA , et al. Preferences for PrEP modalities among gay, bisexual, and other men who have sex with men from Brazil, Mexico, and Peru: a cross‐sectional study. Ther Adv Infect Dis. 2023;10:10:20499361231153548. 10.1177/20499361231153548 PMC9940158 36814515

[16] UNAIDS . About the impact of US funding cuts on the global HIV response: The Timeline. 2025. accessed July 23, 2025. https://www.unaids.org/en/impact‐US‐funding‐cuts/About

[17] The White House . Reevaluating and Realigning United States Foreign Aid. 2025. accessed July 1, 2025. https://www.whitehouse.gov/presidential‐actions/2025/01/reevaluating‐and‐realigning‐united‐states‐foreign‐aid/

[18] US Department of State . PEPFAR: Latest Global Program Results. 2024. accessed July 23, 2025. https://www.state.gov/pepfar‐latest‐global‐results‐factsheet‐dec‐2024/

[19] Brink DT , Martin‐Hughes R , Bowring AL , Wulan N , Burke K , Tidhar T , et al. Impact of an international HIV funding crisis on HIV infections and mortality in low‐ and middle‐income countries: a modelling study. Lancet HIV. 2025;12(5):e346–e354. 40157378 10.1016/S2352-3018(25)00074-8

[20] Tram KH , Ratevosian J , Beyrer C . By executive order: the likely deadly consequences associated with a 90‐day pause in PEPFAR funding. J Int AIDS Soc. 2025;28(3):e26431. 39996580 10.1002/jia2.26431 PMC11851316

[21] Lankiewicz E , Sharp A , Drake P , Sherwood J , Macharia B , Ighodaro M , et al. Early impacts of the PEPFAR stop‐work order: a rapid assessment. J Int AIDS Soc. 2025;28(2):e26423. 39964153 10.1002/jia2.26423 PMC11834162

[22] Hontelez JAC , Goymann H , Berhane Y , Bhattacharjee P , Bor J , Chabata ST , et al. The impact of the PEPFAR funding freeze on HIV deaths and infections: a mathematical modelling study of seven countries in sub‐Saharan Africa. eClinicalMedicine. 2025;83:103233. 40626258 10.1016/j.eclinm.2025.103233 PMC12230335

[23] Cavalcanti DM , de Oliveira Ferreira de Sales L , da Silva AF , Basterra EL , Pena D , Monti C , et al. Evaluating the impact of two decades of USAID interventions and projecting the effects of defunding on mortality up to 2030: a retrospective impact evaluation and forecasting analysis. Lancet. 2025;406(10500):283–94. 40609560 10.1016/S0140-6736(25)01186-9 PMC12274115

[24] Freking K , Jalonick M . Congress approves Trump's $9 billion cut to public broadcasting and foreign aid. Associated Press; 2025. accessed July 23, 2025. https://apnews.com/article/pbs‐npr‐budget‐cuts‐trump‐republicans‐b0044285659ab708e23eb2dc2f3eabfa

[25] Nolen S . U.S. quietly drafts plan to end program that saved millions from AIDS. The New York Times, 2025. accessed July 23, 2025. https://www.nytimes.com/2025/07/23/health/pepfar‐shutdown.html?campaign_id=190&emc=edit_ufn_20250723&instance_id=159089&nl=from‐the‐times&regi_id=170193099&segment_id=202465&user_id=b59ecbe29362453fa08a1064cfd534e2

[26] National Heart L, and Blood Institute NHLBI Funding Compliance Working Group . Award Assessments for Alignment with Agency Priorities. 2025. accessed August 08, 2025. www.science.org/do/10.1126/science.zayz7xq/full/nhlbi‐1749844253710.pdf

[27] Reardon S . Exclusive: NIH document reveal inconsistencies in grant terminations as agency reviews 3200 more. Science. 2025. accessed August 08, 2025. https://www.science.org/content/article/exclusive‐nih‐documents‐reveal‐inconsistencies‐grant‐terminations‐agency‐reviews‐3200

[28] The Global Fund . Government and Public Donors Profiles: United States. 2024. accessed July 20, 2025. https://www.theglobalfund.org/en/government/profiles/united‐states/

[29] The Global Fund . Grants: Americas. 2025. accessed July 23, 2025. https://data.theglobalfund.org/grants?locations=Americas

[30] Friends of the Global Fight . The enduring impact of the 1:2 match requirement. 2025. accessed July 23, 2025. https://www.theglobalfight.org/the‐enduring‐impact‐of‐the‐match‐requirement/

[31] UNAIDS . The critical impact of the PEPFAR funding freeze for HIV across Latin America and the Caribbean. 2025. accessed March 15, 2025. https://www.unaids.org/en/resources/presscentre/featurestories/2025/february/20250219_latin‐america‐caribbean

[32] UNAIDS . Fact Sheet: a snapshot on HIV commodity availability and management risks. 2025. https://www.unaids.org/sites/default/files/2025‐05/UNAIDS_focused‐analysis_HIV‐commodities_en.pdf.

[33] The White House . Defending Women from Gender Ideology Extremism and Restoring Biological Truth to the Federal Government. 2025.

[34] The White House . Protecting American Communities From Criminal Aliens. 2025. accessed May 21, 2025. https://www.whitehouse.gov/presidential‐actions/2025/04/protecting‐american‐communities‐from‐criminal‐aliens/

[35] The White House . Restoring Equality of Opportunity and Meritocracy. 2025. accessed May 21, 2025. https://www.whitehouse.gov/presidential‐actions/2025/04/restoring‐equality‐of‐opportunity‐and‐meritocracy/

[36] UNAIDS . Regional Update: Impact of US Funding Cuts on Global AIDS Response. 2025. https://www.unaids.org/en/resources/presscentre/featurestories/2025/march/20250319_LAC_fs.

